# Role of hypoxia-related genes and immune infiltration in intervertebral disc degeneration: molecular mechanisms and diagnostic potential

**DOI:** 10.3389/fimmu.2025.1606905

**Published:** 2025-07-29

**Authors:** Kai Zhou, Jiaxiang Zhou, XianJin Luo, Yan Chen, Jian Ao, Wei Wu, Bo Yang, Zhongyuan He

**Affiliations:** ^1^ Department of Orthopedics, People's Hospital of Chongqing Liang Jiang New Area, Chongqing, China; ^2^ Deparment of Orthopaedics, the Seventh Affiliated Hospital of Sun Yat-sen University, Shenzhen, China; ^3^ Department of Orthopedics, the Second Affiliated Hospital of Chongqing Medical University, Chongqing, China

**Keywords:** hypoxia, intervertebral disc degeneration, biomarkers, immune infiltration, gene expression

## Abstract

**Objective:**

To investigate the role of hypoxia-related genes and immune infiltration in intervertebral disc degeneration (IDD) to identify molecular mechanisms and potential therapeutic targets.

**Methods:**

Using GEO data, IDD-related gene expression datasets were analyzed for hypoxia-related differentially expressed genes (HRDEGs). Logistic regression and receiver operating characteristic (ROC) analyses were employed to evaluate the diagnostic potential of HRDEGs. Consensus clustering further delineated molecular subtypes of IDD. Functional enrichment analyses (GO, KEGG, GSEA) highlighted key pathways. Protein-protein interaction (PPI) networks were built in STRING and visualized with Cytoscape, identifying core genes with MCODE and CytoHubba. Immune cell infiltration was analyzed with CIBERSORT and ssGSEA to correlate immune cells with hypoxia-related genes. To validate the expression of potential biomarkers, qPCR and immunohistochemistry were conducted on human intervertebral disc tissue samples.

**Results:**

The integration of GSE150408 and GSE124272 datasets with batch effect removal enabled differential gene analysis, identifying nine HRDEGs, including RCOR2, STAT3, and NOTCH1. Logistic regression analysis demonstrated that these genes have high diagnostic efficacy for IDD. Co-expression and clustering analyses revealed two distinct molecular subtypes in IDD, each characterized by unique gene expression and immune infiltration profiles. Functional and pathway enrichment analyses also showed that these DEGs are involved in pathways regulating TP53 transcription, oxidative phosphorylation, and MAPK signaling, contributing to IDD pathology.

**Conclusions:**

Hypoxia-related genes, particularly RCOR2, STAT3, and NOTCH1, play a significant role in the pathology of IDD and may serve as valuable diagnostic biomarkers and therapeutic targets, with distinct immune infiltration patterns associated with different IDD subtypes.

## 1 Introduction

Many patients visit the spine outpatient clinics with low back pain (LBP), which often has a structural cause that cannot be identified. LBP is a major contributor to the global disease burden, with annual healthcare and indirect costs estimated at $100 billion (1–3). Intervertebral disc degeneration (IDD), the leading cause of LBP, is marked by disc structure destruction, nucleus pulposus cell apoptosis, proinflammatory cytokine release, and extracellular matrix degradation (4–6). The functional spinal motion segment consists of the intervertebral disc and adjoining superior and inferior vertebrae, capable of polyaxial movement and withstanding compressive and tensile loads. The motion segment acts as a slow-moving joint, with two hyaline cartilage endplates (CEP) enclosing the nucleus pulposus (NP), which is rich in chondroitin sulfate proteoglycans and confers the disc its swelling properties (7, 8).

The intervertebral disc, recognized as the largest avascular structure in vertebrates, is circumferentially enclosed by the lamellated fibrocartilaginous annulus fibrosus (AF). While the subchondral vasculature perfuses the bony endplate (BEP) and superficial zones of the CEP and AF, it fails to infiltrate the inner AF lamellae or NP parenchyma. This avascularity imposes persistent hypoxia on disc-resident cells (9–11), a microenvironment paradoxically critical for maintaining disc homeostasis through regulation of metabolic adaptation, anabolic matrix biosynthesis, and cellular viability (12–14). Central to hypoxia sensing is the hypoxia-inducible factor (HIF) family-comprising HIF-1α, HIF-2α, and HIF-3α isoforms that dimerize with the constitutively expressed aryl hydrocarbon receptor nuclear translocator (ARNT, HIF-β) (12). Notably, NP cells exhibit constitutive HIF-1α/2α stabilization, enabling hypoxia-tolerant phenotypes via transcriptional coordination of glycolysis and autophagy (15–17). Deciphering this hypoxia-HIF-ECM axis provides mechanistic insights into IDD pathogenesis, positioning HIF modulation as a promising therapeutic strategy to mitigate disc degeneration.

We interrogated transcriptome profiles from the Gene Expression Omnibus (GEO) database to systematically map hypoxia-related differentially expressed genes (HRDEGs) across intervertebral disc tissues and peripheral blood mononuclear cells (PBMCs) in IDD patients versus healthy controls. Through Gene Ontology (GO), Kyoto Encyclopedia of Genes and Genomes (KEGG) pathway analysis, protein-protein interaction (PPI) networks, and other bioinformatics approaches, we identified key pathways and proteins, providing novel insights into the pathophysiology of IDD as well as potential strategies for disc repair and regeneration.

## 2 Materials and methods

### 2.1 Data acquisition

**Figure 1:**
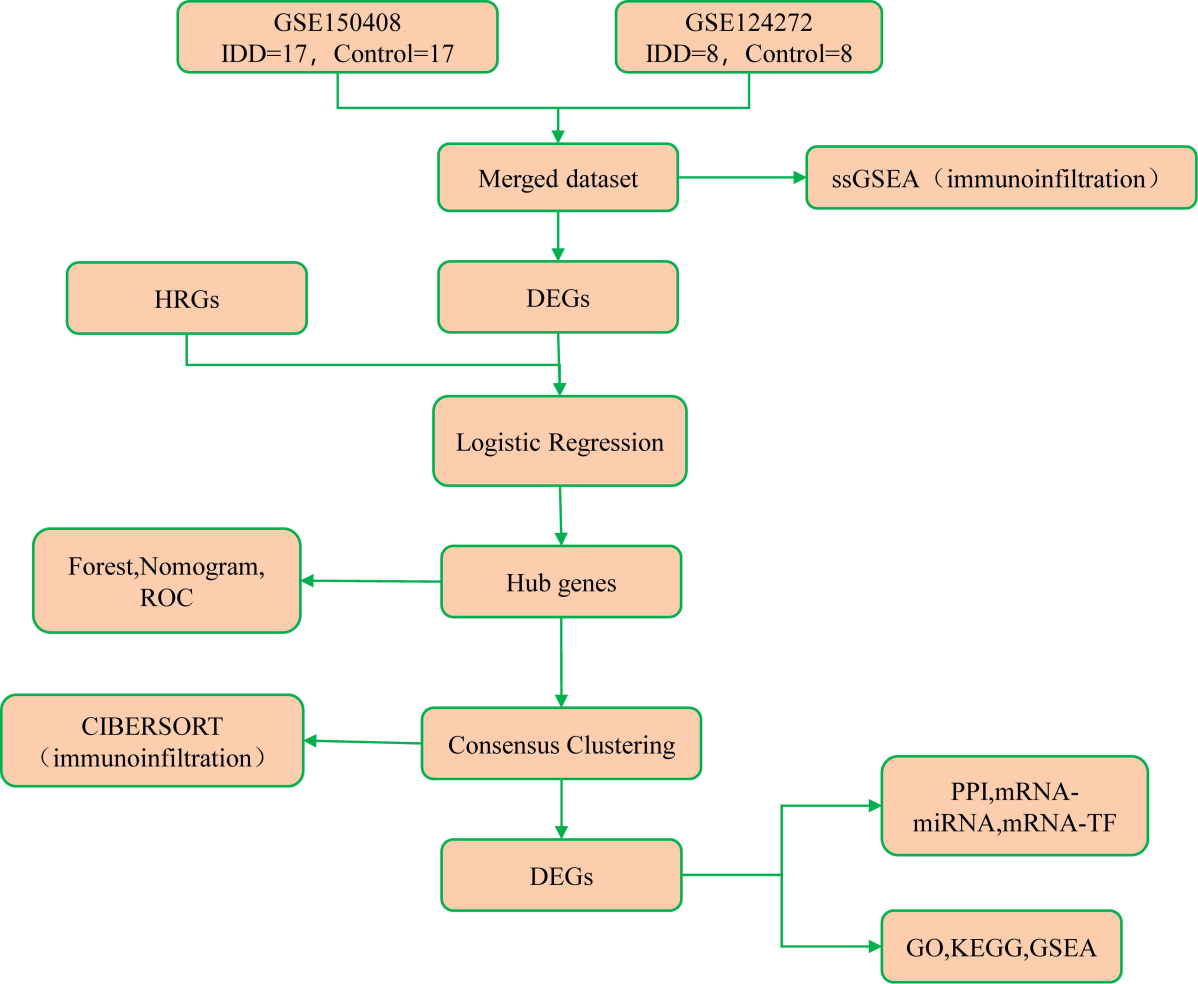
Flow diagram presenting the main plan and process of the study.

This study procedure was conducted methodically based on the steps outlined in the flow diagram (**Figure 1**). Two expression profile datasets, namely GSE150408 (18) (containing 17 IDD cases and 17 controls) and GSE124272 (19) (8 IDD cases and 8 controls), were sourced from the GEO database using the R package GEOquery (**Supplementary Table 1**). These datasets pertain to Homo sapiens and are derived from whole blood tissue. Both the GSE150408 and GSE124272 datasets used Agilent’s microarray platform (GPL21185). Notably, the GSE150408 dataset included 25 samples treated with traditional Chinese medicine, which were excluded from our analysis. Thus, 50 samples (25 intervertebral disc degeneration cases and 25 controls) were included for thorough analysis. Platform annotation files were obtained to convert probe names to gene names to enhance analysis readiness. Additionally, we replaced multiple expression measurements of specific genes with their corresponding mean values. This enabled seamless integration of both datasets, facilitating subsequent in-depth analysis.

### 2.2 Differential gene analysis and hypoxia-related genes

We acquired hypoxia-related genes (HRGs) from GeneCards (https://www.genecards.org/) using “Hypoxia” as the search term and specific filters. This yielded 279 HRGs. We also obtained 50 HRGs from MSigDB’s BUFFA HYPOXIA METAGE.v2022.1. Hs reference gene set using the same keyword. Furthermore, a PubMed search with “Hypoxia” provided 397 HRGs (20). After removing duplicates, we compiled 689 unique HRGs, detailed in **Supplementary Table 5**. Using the data’s grouping (IDD/Control), we employed R’s limma package to identify differentially expressed genes. Those with logFC > 0 and P-value < 0.001 were considered upregulated DEGs, while logFC < 0 and P.adjust < 0.05 signified downregulated DEGs. By intersecting these genes with HRGs, we derived HRDEGs. Similarly, based on data clustering (cluster1/cluster2), we identified differentially expressed genes. Upregulated DEGs had logFC > 1 and P.adjust < 0.05, while downregulated DEGs had logFC < -1 and P.adjust < 0.05.

### 2.3 Logistic regression and phenotypic score

Logistic regression analysis was conducted on hypoxia-related differentially expressed genes to build a logistic regression model. In this context, intervertebral disc degeneration and control were binary dependent variables. Differentially expressed genes relevant to hypoxia were screened with a P value < 0.05 threshold. Using R’s rms package, we constructed a nomogram—a graphical representation of relationships between multiple independent variables—based on logistic regression results. The nomogram illuminated gene contributions to intervertebral disc degeneration. The Receiver Operating Characteristic (ROC) Curve is a valuable tool for selecting the optimal model, determining thresholds, or comparing models. The ROC curve encapsulates sensitivity and specificity relationships through its continuous variables. Using the pROC package in R, we plotted the ROC curve for hypoxia-related genes in intervertebral disc degeneration samples and computed the Area Under the Curve (AUC) to assess the diagnostic efficacy of hub gene expression for intervertebral disc degeneration survival. AUC ranges from 0.5 to 1, with higher values indicating improved diagnostic performance.

### 2.4 Molecular subtyping with phenotypic-related hub genes

Consistency clustering is a resampling-based algorithm that identifies subgroup members and numbers, verifying clustering validity. Using the R package ConsensusClusterPlus, we applied the consensus clustering method to pinpoint intervertebral disc degeneration subtypes using hypoxia-related key genes. Key analysis parameters included maxK = 6, reps = 1000, pItem = 0.8, clusterAlg = ‘km’, and distance = ‘euclidean’.

### 2.5 Functional enrichment analysis (GO) and pathway enrichment (KEGG)

GO and KEGG analyses were performed using the clusterProfiler package. These analyses involved examining differentially expressed genes across various subtypes. We applied entry screening criteria of p.adj < 0.05 and FDR (q value) < 0.05, and corrected using the Benjamini-Hochberg (BH) method, which signifies statistical significance.

### 2.6 Gene Set Enrichment Analysis

Gene Set Enrichment Analysis (GSEA) assesses gene distribution trends within a pre-defined gene set sorted by phenotype correlation, revealing each gene’s contribution to the phenotype. Using the grouping information of Cluster1 and Cluster2 in the dataset, differential analysis was conducted. Subsequently, we utilized R’s clusterProfiler package to perform GSEA on logFC values of all genes in the merged dataset. Parameters included seed 2020, calculation frequency 1000, minimum gene set size of 10 and maximum gene set size of 500. The p-value correction method used was the Benjamini-Hochberg (BH) method. The c2.cp.all.v2022.1.Hs.symbols.gmt gene set was obtained from MSigDB for GSEA (21). Enrichment significance was determined by adj. p < 0.05 and an FDR value (q-value) < 0.05.

### 2.7 Protein-protein interaction network

The PPI network, constructed from interacting proteins, was based on the STRING database. We used this database to build PPI networks related to differentially expressed genes of distinct subtypes, with a minimum required interaction score of 0.400 (medium confidence). The Cytoscape software facilitated PPI network visualization. The MCODE plugin was employed to identify core gene clusters among subtype-specific differentially expressed genes, using thresholds such as Degree Cutoff = 2, Node Score Cutoff = 0.2, K-core = 2, and Max. Depth = 100. Additionally, we utilized CytoHubba’s MCC, DMNC, and MNC algorithms to assess DEG scores related to other PPI network nodes. Based on these scores, we ranked the top 10 DEGs using the three algorithms. The core genes were determined by intersecting these top-ranked DEGs with those from the MCODE analysis.

### 2.8 Construction of mRNA-miRNA and mRNA-TF interaction networks

The CHIPBase database (version 3.0) was utilized to analyze transcription factors’ regulatory effects on core genes. The resulting information was used to visualize mRNA-TF regulatory networks in Cytoscape. For miRNA analysis, starBase 3.0 was used to identify miRNAs associated with core genes. The resulting set was intersected to create miRNA-hub gene relationships. Subsequently, the mRNA-miRNA Regulatory Network was visualized using Cytoscape.

### 2.9 Immunological infiltration analysis

The CIBERSORT algorithm leverages linear support vector regression to estimate immune cell composition and abundance based on transcriptome expression data. Filtering based on p-value < 0.05 yielded the immune cell infiltration matrix. Data with immune cell enrichment scores greater than zero were retained to generate the immune cell infiltration matrix. Correlation heatmaps were drawn using R’s pheatmap to display LM22 immune cell correlations. Single Sample Gene Set Enrichment Analysis (ssGSEA) quantified immune cell infiltration relative abundance. Enrichment scores were calculated for different immune cell types and samples. These fractions represented immune cell infiltration abundance in each sample. Correlation heatmaps were drawn to display the relationship between hypoxia-related hub genes and ssGSEA immune cells.

### 2.10 Real-Time Quantitative Polymerase Chain Reaction

In this study, the intervertebral disc specimens were obtained from human donors undergoing spinal surgery. The degeneration levels of intervertebral discs were classified based on the Pfirrmann grading system using preoperative MRI evaluations. The IDD group included discs classified as Grade III–V, indicating moderate to severe degeneration characterized by decreased disc height, reduced signal intensity, and structural changes in the nucleus pulposus and annulus fibrosus. The control group consisted of discs classified as Grade I–II, representing healthy or mildly degenerated discs with normal disc height and high signal intensity on MRI. All donors were carefully screened to exclude individuals with systemic inflammatory diseases, autoimmune disorders, metabolic bone diseases, tumors, or active infections to ensure the health status of the specimen source hosts. The collected disc tissues were used for RNA extraction, gene expression analysis, histological staining, and immunohistochemistry.

Total RNA was extracted from NP tissues following established protocols. Briefly, 150 mg of tissue was digested with 2 mg/mL pronase at 37°C, flash-frozen, pulverized in liquid nitrogen, and homogenized using a tissue lyser (Qiagen/Retsch^®^, Germany). RNA was isolated with TRI Reagent (Invitrogen, United States), and 400 ng was reverse transcribed to cDNA using a synthesis kit (Takara, Japan). RNA concentration was measured with a spectrophotometer (NanoDrop™ One/2000, United States), and a 20μL reaction mixture was prepared with 400 ng RNA, 4μL of reaction mix, and DEPC water (Beyotime, China), followed by reverse transcription on a PCR instrument (Bio-Rad, United States). RT-qPCR was performed using qPCR Mix (Thermo Fisher Scientific, United States) on a CFX96 system (Bio-Rad, United States). Each 10 µL reaction contained 5 µL of Fast SYBR Green master mix, 2 μL RNase-free ddH_2_O, 2 μL cDNA, and 0.5 μL of each primer (**Supplementary Table 2**). Relative expression was calculated by the 2−ΔΔCt method, normalized to GAPDH.

### 2.11 Histological staining

Patients’ samples were fixed in 4% paraformaldehyde for 24 hours at 4°C and then decalcified in an EDTA solution at the same temperature. Subsequently, the samples were embedded in paraffin and sectioned at a thickness of 6μm. Routine hematoxylin and eosin (H&E) and Safranin O/Fast Green (SOFG) staining were performed, followed by image capture. For immunohistochemistry (IHC), sections were deparaffinized, rehydrated, and subjected to antigen retrieval using citrate buffer (pH 6.0). After blocking, samples were incubated overnight at 4°C with primary antibodies targeting RCOR2 and NOTCH1, followed by DAB staining and hematoxylin counterstaining. For immunofluorescence staining, samples were permeabilized in 100 mL of 0.3% Triton X-100 (Sigma, T8787) solution in 1% PBS at room temperature for 30 minutes. Blocking was then performed with 5% BSA (Biofroxx, 4240GR100) and 0.1% Triton X-100 solution. After the blocking solution was removed, the samples were incubated overnight at 4°C with primary antibodies targeting STAT3 and p-STAT3. The following day, samples were incubated for 1 hour with secondary antibodies, specifically donkey anti-rabbit IgG (H+L), highly cross-adsorbed secondary antibody, and Alexa Fluor Plus 594. Finally, the samples were counterstained with DAPI. Images were captured using a Leica DM6B microscope and a Zeiss LSM 880 confocal microscope.

### 2.12 Statistical analysis

All data calculations and statistical analyses were conducted using R (version 4.2.0, https://www.r-project.org/). Independent Student’s t-test was used to estimate significance for normally distributed variables, while non-normally distributed variables were analyzed using the Mann-Whitney U-test (Wilcoxon rank-sum test). All statistical P-values were two-tailed, with P < 0.05 considered statistically significant.

## 3 Results

### 3.1 Data preprocessing

The SVA package in R was used to eliminate batch effects from the two IDD datasets, GSE150408 and GSE124272. Following batch effect removal, the resulting merged dataset (mdata) was standardized using the Limma package, producing the merged dataset (**Supplementary Figures 1A, B**). Comprising 25 IDD samples (Group: IDD) and 25 control samples (Group: Control), the Merged dataset was subjected to Principal Component Analysis (PCA) both before and after batch effect removal, based on sample source (**Supplementary Figures 1C, D**). The PCA results confirmed that batch effects were effectively eliminated in the merged dataset.

### 3.2 Differential analysis and gene similarity analysis

**Figure 2:**
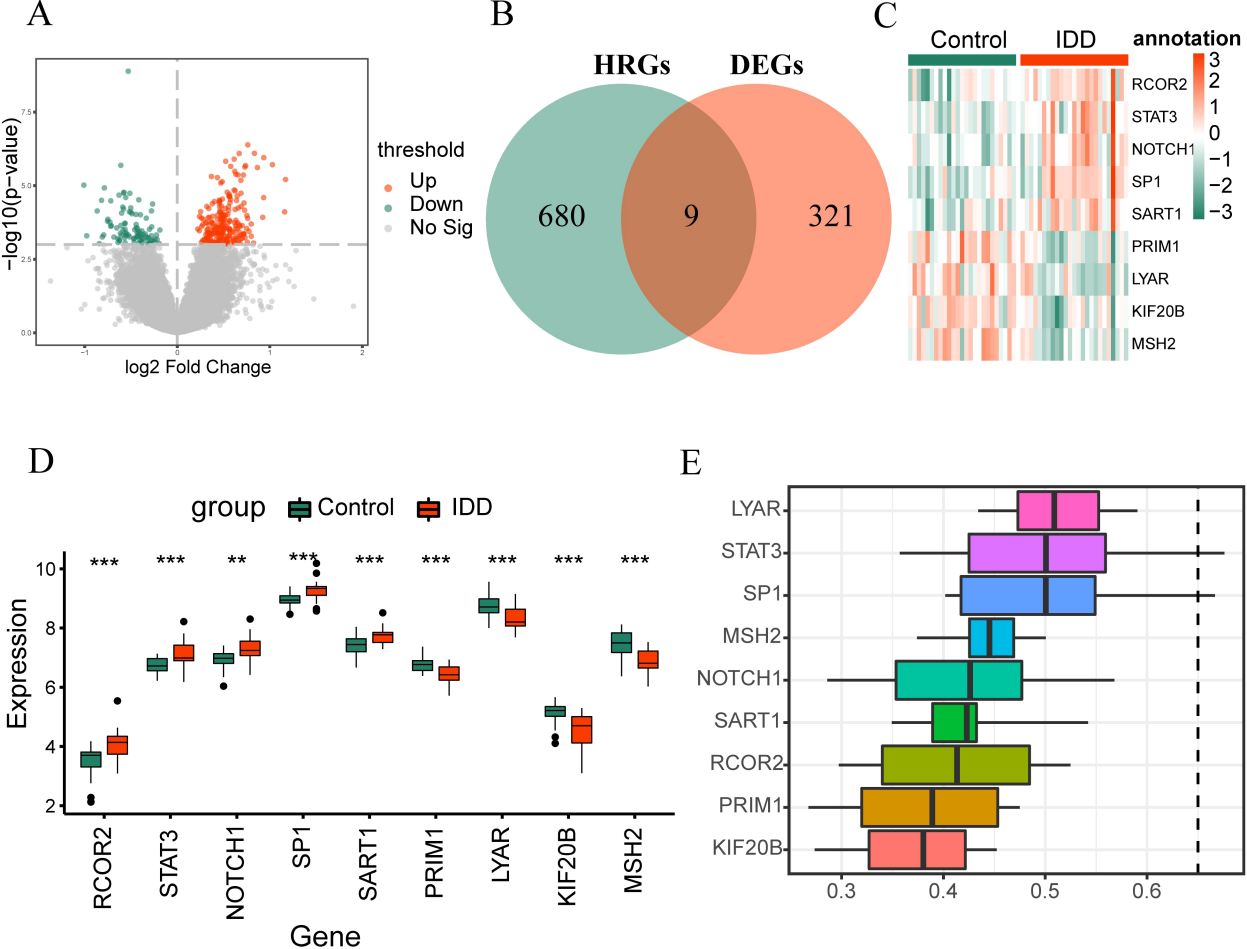
Differential analysis and gene similarity analysis. **(A)** Illustrates a volcano plot displaying differentially expressed genes between the IDD group and Control group (Control) in the Merged dataset. **(B)** Venn plot showcasing the overlap between differentially expressed genes and hypoxia-related genes. **(C)** Shows a complex numerical heatmap detailing the HRDEGs within the Merged dataset. **(D)** Compares the grouping of RCOR2, STAT3, NOTCH1, SP1, SART1, PRIM1, LYAR, KIF20B, and MSH2 between IDD and Control groups. **(E)** Presents results of functional similarity analysis for RCOR2, STAT3, NOTCH1, SP1, SART1, PRIM1, LYAR, KIF20B, MSH2. *p < 0.05; **p < 0.01; ***p < 0.001.

Differential analysis was conducted using the Limma package to identify expression differences between the IDD and Control groups within the merged dataset. Out of 34,730 differentially expressed genes, 330 met the criteria of | logFC |>0 and p-value<0.001. The subset included 232 upregulated genes (positive logFC) in IDD and 98 downregulated genes (negative logFC) in IDD. Differential analysis results were visualized with a volcano plot (**Figure 2A**). Subsequently, we identified HRDEGs by intersecting differentially expressed genes with hypoxia-related genes, yielding nine genes: RCOR2, STAT3, NOTCH1, SP1, SART1, PRIM1, LYAR, KIF20B, and MSH2 (**Figure 2B**). The expression patterns of these genes were depicted via an expression heatmap (**Figure 2C**), showcasing their distinctive profiles between the IDD and Control groups.

We performed Wilcoxon rank-sum tests to compare the expression of these nine genes between IDD and Control groups. Results displayed in the group comparison graph confirmed statistically significant expression differences (p < 0.05) between the two groups (**Figure 2D**). The expression of RCOR2, STAT3, NOTCH1, SP1, and SART1 was elevated in IDD, while PRIM1, LYAR, KIF20B, and MSH2 exhibited lower expression levels in IDD.

Next, we performed a functional similarity analysis of the nine genes. We calculated semantic similarity between GO terms and gene products using the GOSemSim package to determine the functional relationships. The functional similarity values were visualized in a boxplot, which revealed that LYAR exhibited the highest functional similarity with the other genes (**Figure 2E**).

### 3.3 Logistic regression

**Figure 3:**
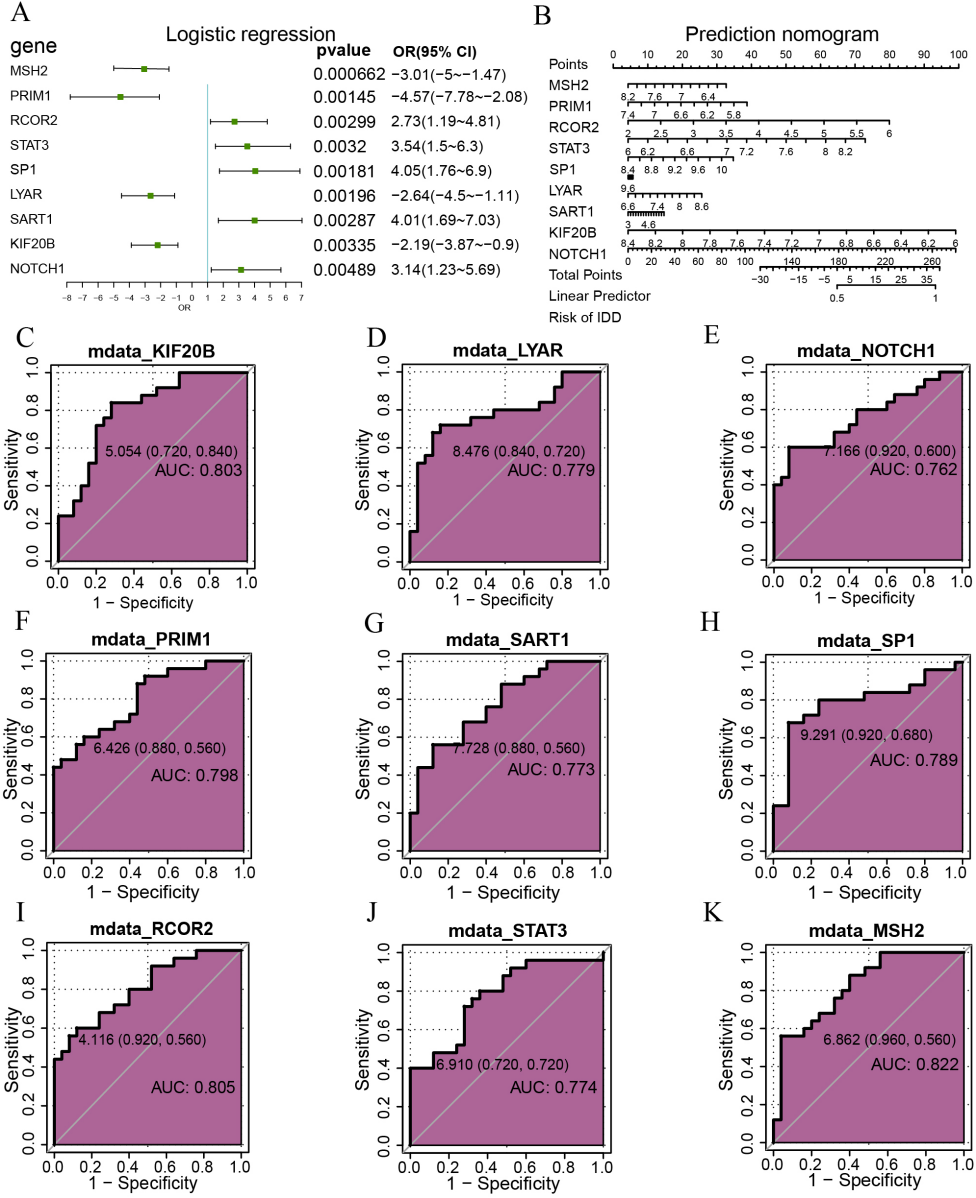
Logistic regression analysis. **(A)** Depicts a forest plot of single-factor logistic regression analysis for hypoxia-related genes within the merged dataset. **(B)** Presents a bar chart displaying RCOR2, STAT3, NOTCH1, SP1, SART1, PRIM1, LYAR, KIF20B, MSH2 genes. **(C-K)** Illustrates ROC curves for specific genes: KIF20B **(C)** LYAR **(D)** NOTCH1 **(E)** PRIM1 **(F)** SART1 **(G)** SP1 **(H)** RCOR2 **(I)** STAT3 **(J)** and MSH2 **(K)** Mdata, Merged Data; ROC, Receiver Operating Characteristic. AUC ranges between 0.5 and 1. AUC close to 1 indicates a better diagnostic effect. AUC below 0.7 suggests lower accuracy, while AUC between 0.7 and 0.9 indicates moderate accuracy, and AUC above 0.9 signifies higher accuracy.

Logistic regression based on the nine hypoxia-related differentially expressed genes was displayed with a forest plot (**Figure 3A**). Nine genes were screened with a p-value<0.05: RCOR2, STAT3, NOTCH1, SP1, SART1, PRIM1, LYAR, KIF20B, MSH2. Nomograms were drawn based on the nine hypoxia-related differentially expressed genes to show the results of the nomogram analysis (**Figure 3B**). The results showed that RCOR2, STAT3, and NOTCH1 expression levels were significantly more effective for diagnosing intervertebral disc degeneration than other variables, while the utility of SP1, SART1, PRIM1, LYAR, KIF20B, and MSH2 expression in diagnosing intervertebral disc degeneration was lower. The utility of SP1, SART1, PRIM1, LYAR, KIF20B, MSH2 expression in the diagnosis of intervertebral disc degeneration was significantly lower than that of other variables. Subsequently, we plotted the ROC curves of nine genes in the combined dataset and displayed the results (**Figures 3C–K**). The results showed that the genes KIF20B (AUC = 0.803, **Figure 3C**), LYAR (AUC = 0.779, **Figure 3D**), NOTCH1 (AUC = 0.762, **Figure 3E**), PRIM1 (AUC = 0.798, **Figure 3F**), SART1 (AUC = 0.773, **Figure 3G**), SP1 (AUC = 0.789, **Figure 3H**), RCOR2 (AUC = 0.805, **Figure 3I**), STAT3 (AUC = 0.774, **Figure 3J**) and MSH2 (AUC = 0.822, **Figure 3K**) expression had certain accuracy in the diagnosis of the occurrence of disc degeneration.

### 3.4 Correlation analysis of hub genes

**Figure 4:**
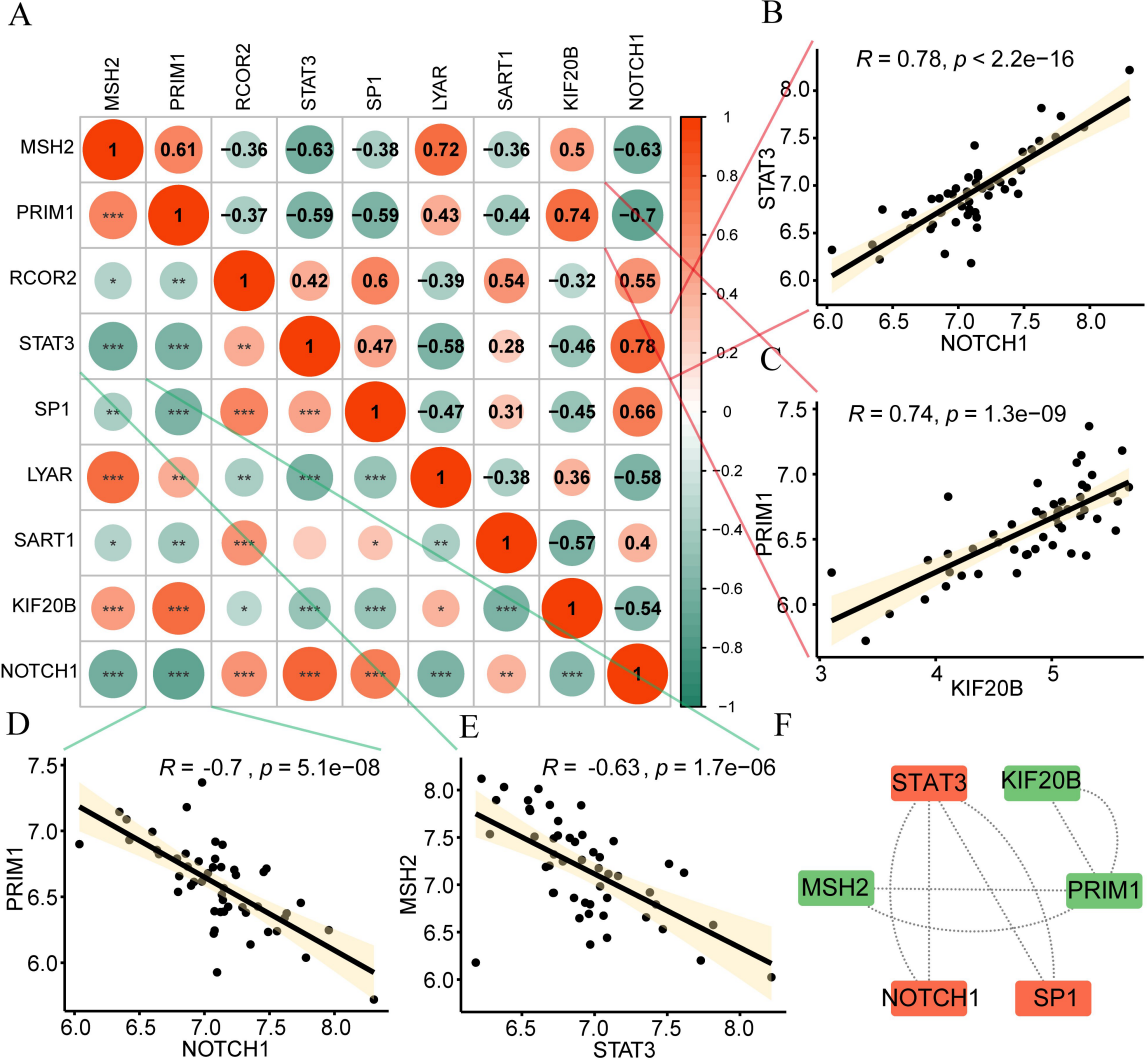
Correlation of hypoxia genes. **(A)** Positive (orange) and negative (green) correlations among hypoxia genes are visualized in the heatmap. **(B-E)** Strong Gene Correlations. Scatter plots depict strong correlations between gene pairs: **(B)** STAT3 and NOTCH1 **(C)** PRIM1 and KIF20B **(D)** PRIM1 and NOTCH1 **(E)** MSH2 and STAT3. **(F)** An interaction network diagram illustrates connections between hub genes. Absolute correlation coefficient (r) values above 0.8 indicate strong correlation; 0.5-0.8, moderate; 0.3-0.5, weak; below 0.3, minimal correlation. ns, no significance; *p < 0.05; **p < 0.01; ***p < 0.001.

Next, Spearman’s method was used to calculate correlations between hypoxia-related genes (**Figure 4A**) and separately displayed the gene relationships with strong correlations (positive and negative top 2). According to the figure, STAT3 and NOTCH1 (R=0.78, p<2.2e-16, **Figure 4B**), PRIM1 and KIF20B (R=0.74, p=1.3e-09, **Figure 4C**) had strong positive correlations. PRIM1 and NOTCH1 (R=-0.7, p=5.1e-08, **Figure 4D**), MSH2 had a strong negative correlation with STAT3 (R=-0.63, p=1.7e-06, **Figure 4E**). In addition, we used the STRING database to perform protein-protein interaction analysis (minimum required interaction score: medium confidence (0.400)) on 9 hub genes. After excluding DEGs without connections to other nodes, we constructed a PPI network with six-node DEGs (**Figure 4F**). Orange and green respectively indicate two gene sets with interaction relationships. STAT3 interacts with NOTCH1 and SP1, and KIF20B interacts with PRIM1 and MSH2.

### 3.5 Consistency clustering analysis of hub genes

**Figure 5:**
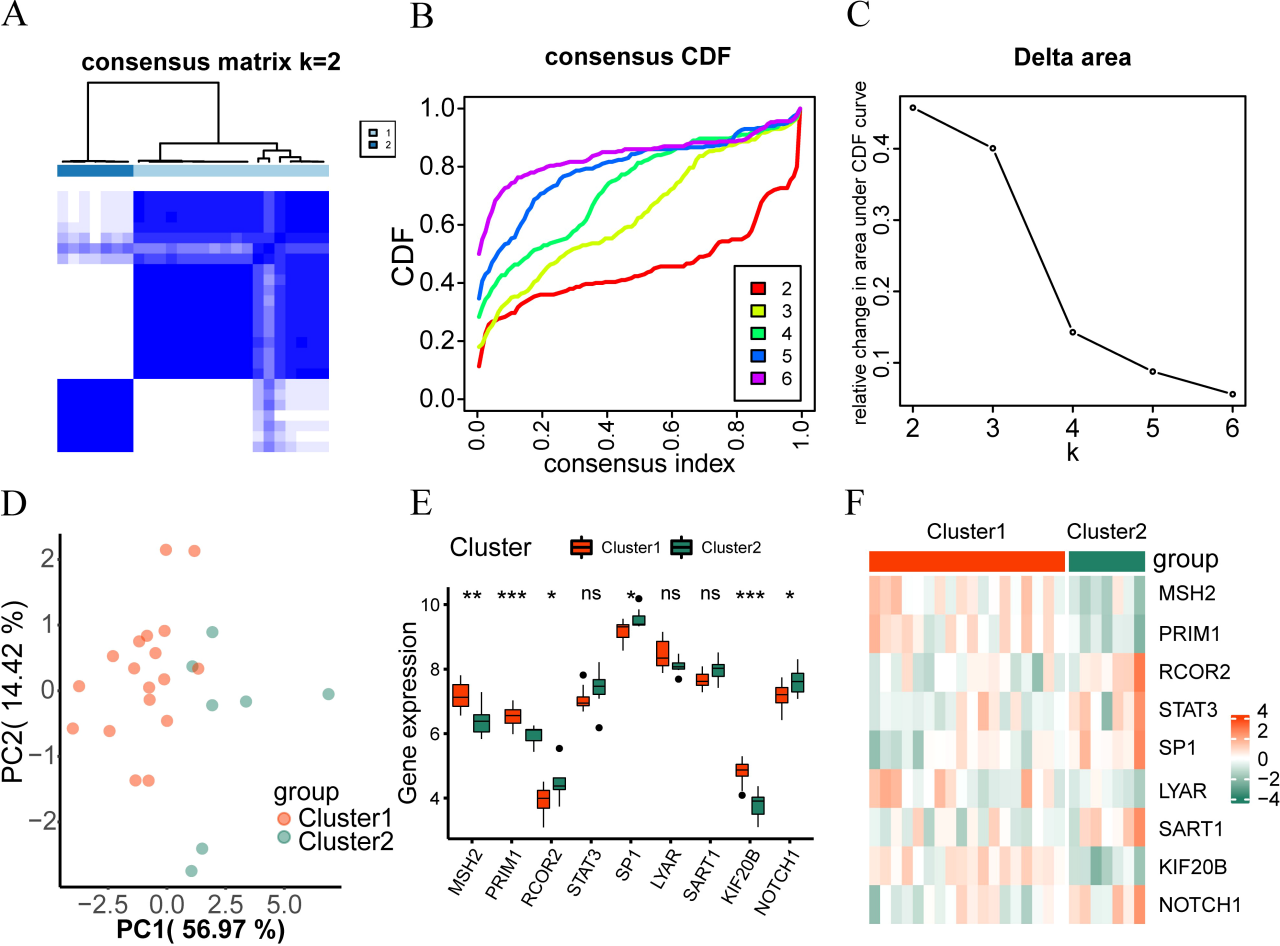
Consistency clustering analysis. **(A)** The clustering result graph (K=2) depicts intervertebral disc degeneration subtypes. **(B, C)** Consistency Clustering Cumulative Distribution Function, **(B)** and Area Delta Diagram **(C)** under CDF Curve for Different Clusters. **(D)** Subtype PCA Analysis, PCA analysis results of cluster1 and cluster2 in the intervertebral disc degeneration dataset. **(E)** Comparison of hub genes in different subtypes of intervertebral disc degeneration. **(F)** Heatmap shows hub gene expression across intervertebral disc degeneration subtypes. ns, no significance; *p < 0.05; **p < 0.01; ***p < 0.001.

To examine the expression differences of hub genes in intervertebral disc degeneration samples, we used the R package “ConsensusClusterPlus” to identify different subtypes related to intervertebral disc degeneration based on the expression of nine hub genes (RCOR2, STAT3, NOTCH1, SP1, SART1, PRIM1, LYAR, KIF20B, MSH2) in the merged dataset, and finally identified two subtypes: Cluster 1 and Cluster 2 (**Figure 5A**). Cluster 1 contained 18 samples, and Cluster 2 contained 7 samples. The Cumulative Distribution Function (CDF) diagram (**Figure 5B**) and the area under the CDF curve delta diagram (**Figure 5C**) illustrate different cluster numbers in the consistent clustering results. **Figure 5C** shows that when k=2, the clustering results for the intervertebral disc degeneration dataset are optimal. Then we performed PCA on the dataset expression matrix of the two subtype samples in the disc degeneration dataset, PCA clustering results showed a clear distinction between the two subtype samples (**Figure 5D**).

The Wilcoxon rank sum test was used to analyze differences in the expression levels of the nine hub genes between Cluster 1 and Cluster 2. **Figure 5** showed statistically significant differences (P < 0.05) except for STAT3, LYAR, and SART1, the expression levels of PRIM1, KIF20B, and MSH2 were higher in disc degeneration subtype 1 (Cluster 1) than in subtype 2 (Cluster 2). However, the expression of RCOR2, NOTCH1, and SP1 in cluster1 was lower than that in cluster2 (up-regulated in cluster2). Finally, we used the ‘pheatmap’ package in R to generate a heatmap showing the expression differences of the nine hub genes in the two-disc degeneration subtypes (**Figure 5F**).

### 3.6 PPI interaction network, mRNA-miRNA, mRNA-TF network construction

**Figure 6:**
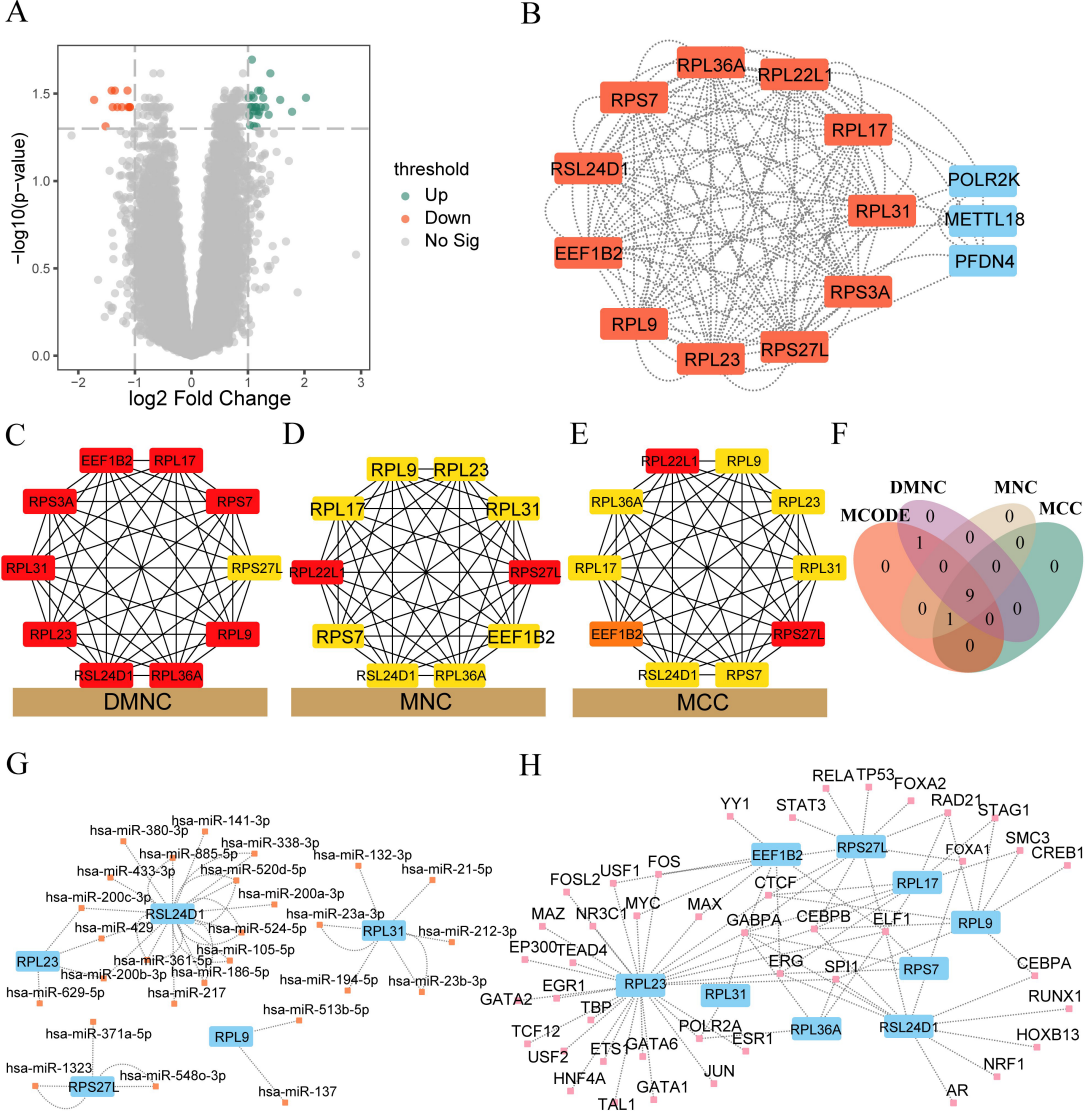
PPI interaction network, mRNA-miRNA, mRNA-TF network construction. **(A)** Volcano plot depicting the differential analysis outcomes between the cluster 1 and cluster 2 groups within the IDD dataset. **(B)** PPI network illustrating the differentially expressed genes (DEGs) between cluster 1 and cluster 2 in the IDD dataset. The core gene clusters recognized by the MCODE algorithm are highlighted in red. **(C-E)** The PPI network visualizes the top 10 genes obtained from DMNC, MNC, and MCC algorithms. The intensity of the color signifies the score obtained from each algorithm. **(F)** Venn diagram showcasing the intersection of the results from four algorithms—MCODE, MCC, DMNC, and MNC—within the PPI network. **(G)** Network illustrating the interaction between hub genes and miRNAs. Hub genes are indicated in blue, while miRNAs are represented in orange. **(H)** Network depicting the interaction between hub genes and TFs. Hub genes are denoted in blue, while TFs are highlighted in pink.

To analyze the difference in gene expression in two different subtypes (Cluster1 and Cluster2) of the IDD group, we used the Limma package to perform differential expression analysis between different subtypes. The results are as follows: a total of 34,730 differentially expressed genes were obtained among different subtypes of samples in the IDD group, of which 39 genes met the threshold of |logFC|>1 and p.adjust<0.05. Under this threshold, 28 genes were highly expressed in the Cluster 1 group (with low expression in the Cluster 2 group, where logFC was positive, indicating up-regulated genes) and 11 genes were lowly expressed in the Cluster 1 group (with high expression in the Cluster 2 group, where logFC was negative). We plotted the difference analysis results of samples in the IDD group using a volcano plot (**Figure 6A**).

We used the STRING database to perform PPI analysis (minimum required interaction score: medium confidence (0.400)) on 39 DEGs. After excluding DEGs without connection with other nodes, we constructed a PPI network composed of 14 DEGs (**Figure 6B**). See **Supplementary Table 6** for specific PPI network node information. The red color indicates the core gene cluster identified by the MCODE plug-in. The core gene cluster contains 11 genes (RPL31, RPL36A, RPS27L, RPS3A, RSL24D1, RPL23, RPL22L1, RPL17, RPL9, EEF1B2, RPS7), with a score of 11.0. At the same time, the DMNC algorithm (**Figure 6C**), MNC algorithm (**Figure 6D**), and MCC algorithm (**Figure 6E**) of the CytoHubba plug-in were used to obtain the top 10 genes. The core gene cluster obtained by MCODE was intersected with the genes obtained by different algorithms of CytoHubba plug-in to obtain nine core genes (RPL31, RPL36A, RPS27L, RSL24D1, RPL23, RPL17, RPL9, EEF1B2, RPS7, **Figure 6F**).

Subsequently, we used the mRNA-miRNA data in the StarBase database to predict the miRNAs interacting with nine core genes (RPL31, RPL36A, RPS27L, RSL24D1, RPL23, RPL17, RPL9, EEF1B2, RPS7), and only retained the mRNA miRNA data pairs common to at least three databases including PITA, RNA22, miRMap, MicroT, miRanda, PicTar, and TargetScan. We then mapped the mRNA-miRNA interaction network using Cytoscape software for visualization (**Figure 6G**). The blue color in the mRNA miRNA interaction network represents mRNA, while orange indicates miRNA. According to the mRNA-miRNA interaction network, our network consists of 5 mRNAs (RPL23, RPL31, RPL9, RPS27L, RSL24D1) and 27 miRNA molecules, constituting a total of 30 pairs of mRNA miRNA interactions. See **Supplementary Table 7** for the specific mRNA-miRNA interactions.

We searched the ChipBase database (version 3.0) for transcription factors (TFs) that bind to core genes. Finally, we obtained the interaction data of 9 mRNAs and 43 TFs, which were visualized using Cytoscape software. The blue color in the mRNA TF interaction network represents mRNA, while the pink color represents TFs (**Figure 6H**). The specific mRNA TF interaction relationships are shown in **Supplementary Table 8**.

### 3.7 Functional enrichment analysis (GO), pathway enrichment analysis (KEGG), and GSEA of differential genes in different subtypes of the IDD group

**Figure 7:**
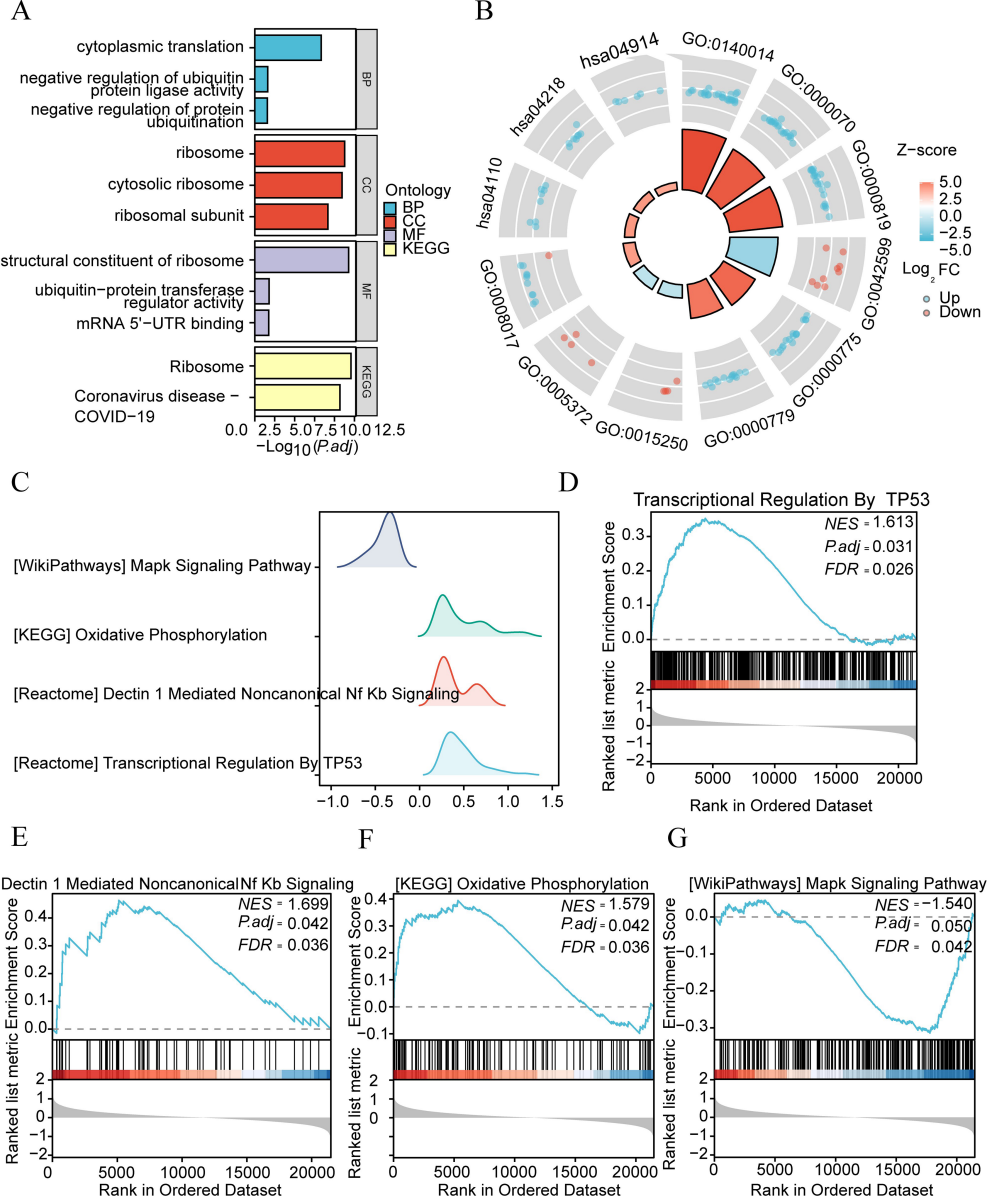
Functional GO and KEGG analysis of differential genes in different subtypes of IDD group. **(A)** Bar chart illustrating GO enrichment analysis and KEGG pathway enrichment analysis outcomes of differential genes among different subtypes in the IDD group. **(B)** Cycle diagram depicting GO and KEGG enrichment pathways. The inner circle’s columns correspond to entries, with height indicating the relative size of p.adj. The filling color represents the zscore value. **(C)** GSEA enrichment analysis of genes among different subtypes in the IDD group. Four biological characteristics are displayed in a mountain plot. **(D-G)**. Significant gene enrichments in IDD group: Transcriptive Regulation by TP53 **(D)** Dectin 1 Mediated Nonocal Nf Kb Signaling **(E)** Oxidative Photosynthesis **(F)** and Mapk Signaling Pathway **(G)**.

To analyze the biological processes, molecular functions, cellular components, and biological pathways related to 39 DEGs, we first performed GO and KEGG enrichment analysis on DEGs. DEGs are mainly enriched in biological processes (BP) such as negative regulation of protein ubiquitination, negative regulation of ubiquitin-protein ligase activity, cytoplasmic translation, ribosomal subunit, cytosolic ribosome, ribosome, and other cellular components (CC), mRNA 5’−UTR binding, ubiquitin-protein transfer regulator activity, structural molecular function (MF) of the ribosome, and other biological pathways such as ribosome and coronavirus disease (COVID-19) (**Figure 7A**), see **Supplementary Table 3**. **Figure 7B** shows the expression of genes related to GO function-enriched pathways and KEGG-enriched pathways, with blue indicating downregulation and red indicating upregulation.

We analyzed the relationship between gene expression and biological processes involved, cellular components affected, and molecular functions exerted using GSEA (Gene Set Enrichment Analysis) (**Figure 7C**), with p.adjust <0.05 and FDR value (q-value) < 0.05 as the significant enrichment screening criteria. The results showed that genes in the merged dataset were significantly enriched in transcriptional regulation by TP53 (**Figure 7D**), dectin 1-mediated non-canonical NF-κB signaling (**Figure 7E**), (KEGG) oxidative phosphorylation (**Figure 7F**), (WikiPathways) MAP signaling pathway (**Figure 7G**), and other pathways (**Supplementary Table 4**).

### 3.8 CIBERSORT immune infiltration analysis

**Figure 8:**
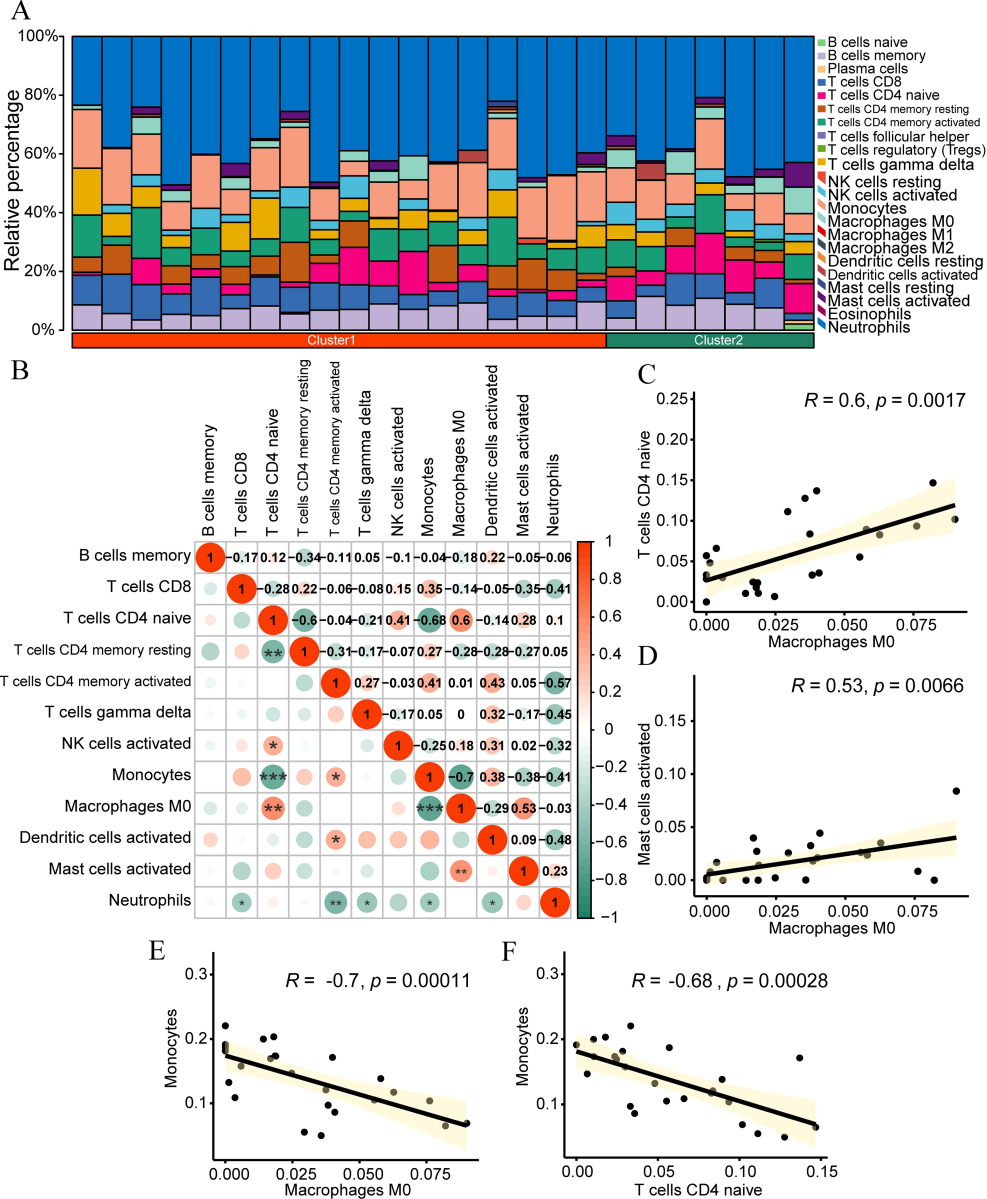
CIBERSORT immune infiltration analysis. **(A)** Stacked bar chart presenting the CIBERSORT immune infiltration analysis results of the IDD group dataset. **(B)** Correlation analysis outcomes of immune cell infiltration abundance in the IDD group dataset. **(C-F)** Scatter plots illustrating the correlation between different immune cells: Macrophages M0 and Naive CD4^+^ T cells **(C)** Mast cells activated and Monocytes **(D)** Macrophages M0 and Monocytes **(E)** and Naive CD4^+^ T cells and Mast cells activated **(F)**. *p < 0.05; **p < 0.01; ***p < 0.001.

To explore the differences in immune infiltration in the samples of the dataset, we used the CIBERSORT algorithm to calculate the abundance of infiltration of 22 kinds of immune cells for the samples of the IDD dataset. Subsequently, we employed a bar plot to illustrate the proportion of immune cell infiltration abundance in the samples of the IDD group (**Figure 8A**). According to the figure, the proportion of neutrophils in the samples of the IDD dataset is relatively high.

We selected the cells with infiltration abundance in at least half of the samples for correlation analysis, including T B cells memory, T cells CD8, Naive CD4^+^ T cells, T cells CD4 memory resetting, T cells CD4 memory activated, T cells gamma delta, NK cells activated, monocytes, macrophages M0, dendritic cells activated, mast cells activated, and neutrophils. Subsequently, we calculated the correlation between the infiltration abundance of these 12 immune cells in the IDD dataset and displayed the results (**Figure 8B**). The results showed that immune cells macrophages M0 had a significant positive correlation with Naive CD4^+^ T cells (r=0.6, p<0.01, **Figure 8C**) and mast cells activated (r=0.53, p<0.01, **Figure 8D**), while monocytes had a significant negative correlation with macrophages M0 (r=-0.7, p<0.01, **Figure 8E**) and Naive CD4^+^ T cells (r=-0.68, p<0.01, **Figure 8F**).

### 3.9 Immunological characteristic typing

**Figure 9:**
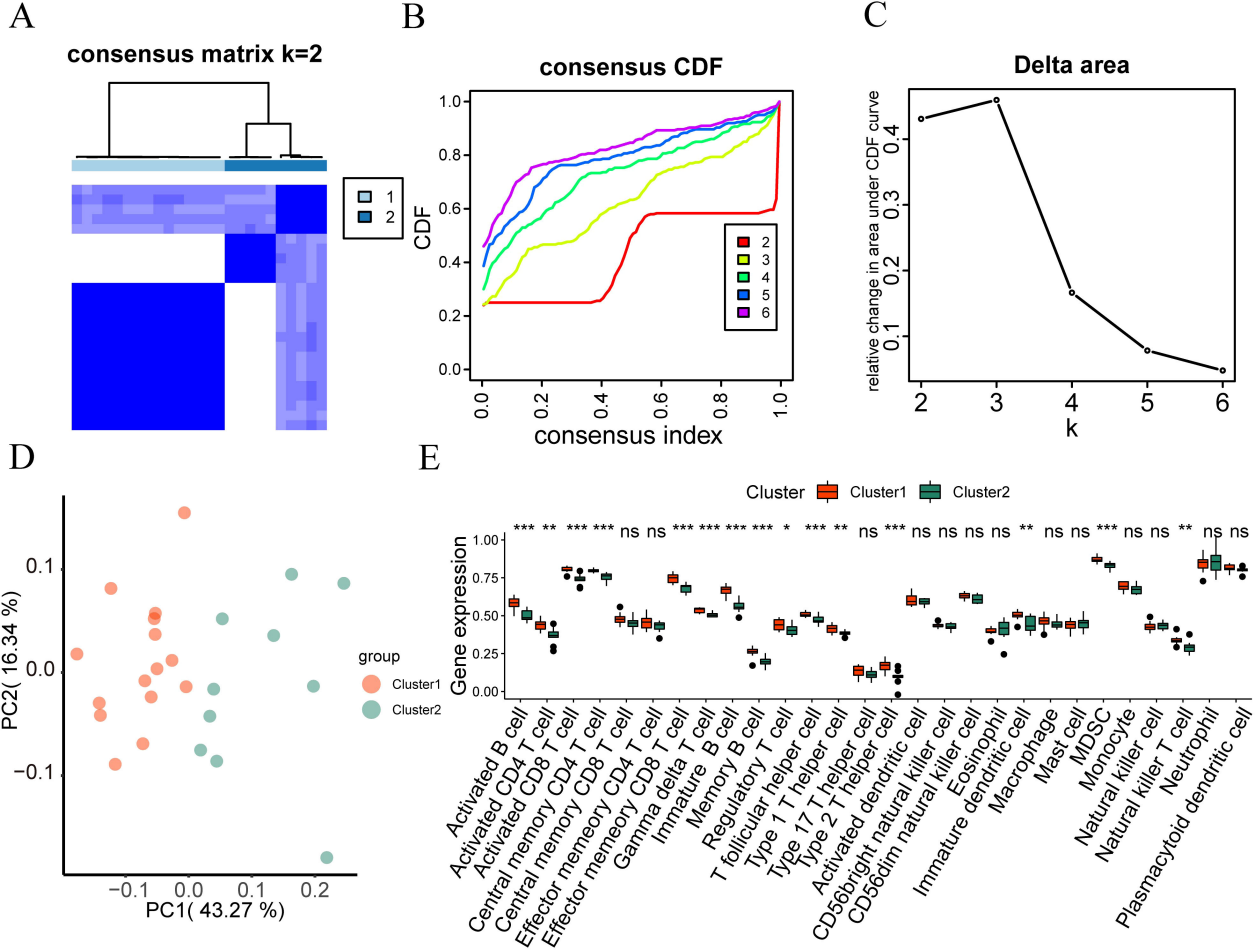
Immunological feature typing. **(A)** Consistency clustering (K=2) results graph of immune infiltration abundance in the IDD group. **(B, C)** Cumulative distribution function and area Delta under the CDF curve for different cluster numbers in consistency clustering. **(D)** PCA analysis results of two immune characteristic subtypes (cluster 1 and cluster 2) in the IDD dataset. **(E)** Grouping comparison of immune cells in different subtypes of immune characteristics in the IDD dataset. ns, no significance; *p < 0.05; **p < 0.01; ***p < 0.001.

To investigate the differences in immune infiltration in IDD, we used the ssGSEA algorithm to analyze the abundance of immune cell infiltration in the samples. We used the R package “ConsensusClusterPlus” and, based on the abundance of immune infiltration, identified different immune feature subtypes related to intervertebral disc degeneration using the consistency clustering method. Finally, two immune feature subtypes (Cluster 1 and Cluster 2) were determined (**Figure 9A**). Immune characteristic subtype 1 (Cluster 1) contains 15 samples, while immune characteristic subtype 2 (Cluster 2) contains 10 samples. We also presented the CDF graph of consistency clusters with different numbers of clusters in the consistency clustering results (**Figure 9B**) and the area under the CDF curve (**Figure 9C**). From the graph, it can be seen that when k=2 is used as the number of unsupervised clusters, the consistency clustering results of the intervertebral disc degeneration dataset are the best. Subsequently, we performed PCA on the expression matrices of two immune feature subtypes in the intervertebral disc degeneration dataset. The PCA clustering results showed that the two immune feature subtypes in intervertebral disc degeneration could be clearly distinguished from each other (**Figure 9D**).

In addition, we also used the Wilcoxon rank-sum test to analyze the difference between the expression levels of 28 immune cells in the intervertebral disc degeneration dataset and the two immune characteristic subtypes. According to **Figure 9E**, the expression of 28 immune cells between the two subtypes of the intervertebral disc degeneration dataset is shown in activated B cell, activated CD4 T cell, activated CD8 T cell, central memory CD4 T cell, effector memory CD8 T cell, gamma delta T cell, image B cell, memory B cell, regulatory T cell, T follicular helper cell, type 1 T helper cell, type 2 T helper cell, image dendritic cell, MDSC, and natural killer T cell, showing statistically significant differences (P < 0.05).

### 3.10 Correlation analysis between Hub genes and immune cells

**Figure 10:**
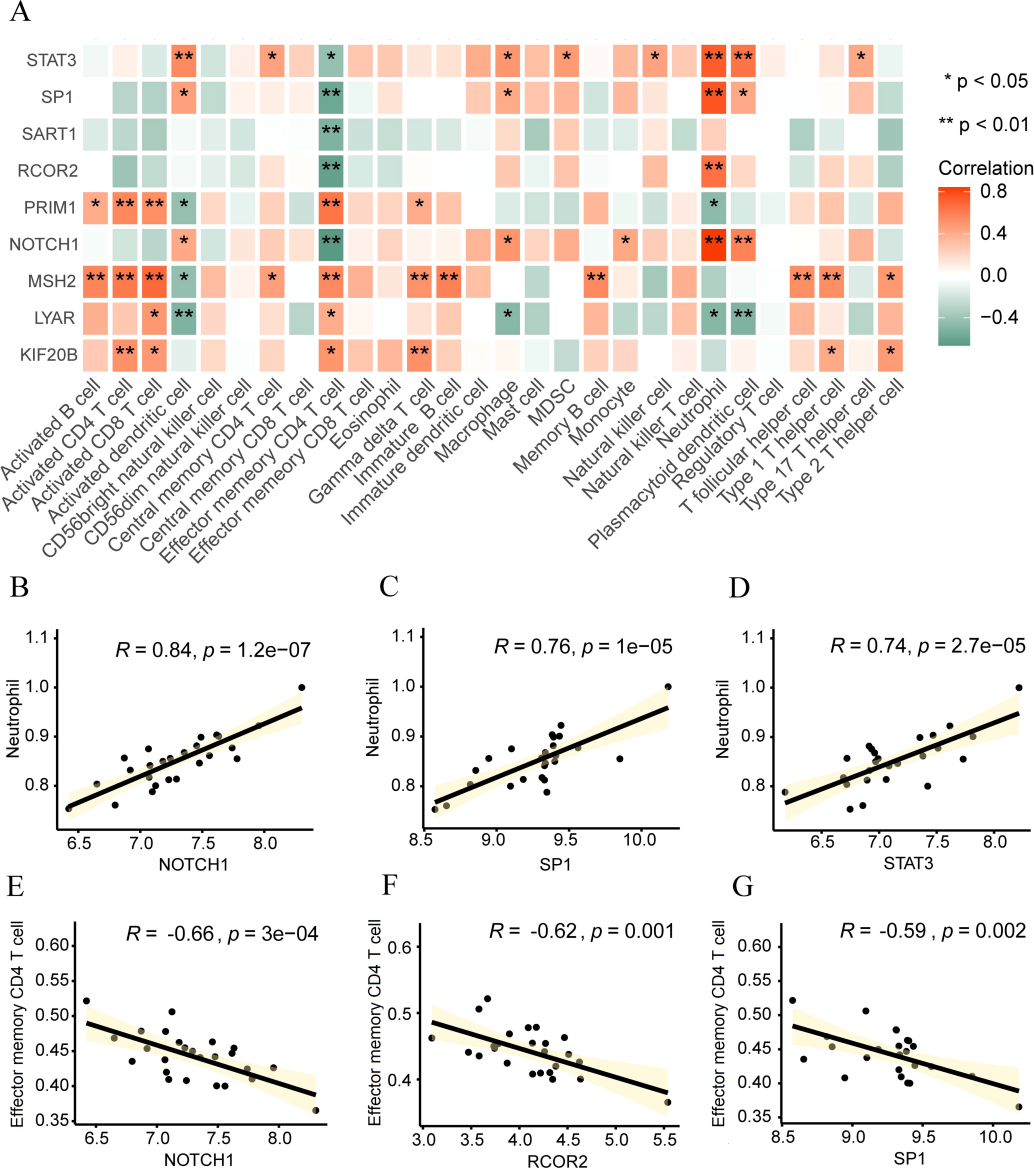
Correlation analysis between Hub genes and immune cells. **(A)** Heat map displaying the correlation between hub genes and immune cells. **(B-G)** Scatter plots illustrating the correlation between immune cells and hub genes: Neutral and NOTCH1 **(B)**, SP1 **(C)**, and STAT3 **(D)**. Effect of memory CD4 T cells on NOTCH1 **(E)**, RCOR2 **(F)**, and SP1 **(G)**. *p < 0.05; **p < 0.01.

To analyze the biological connection between hypoxia-related hub genes (RCOR2, STAT3, NOTCH1, SP1, SART1, PRIM1, LYAR, KIF20B, MSH2) and the immune microenvironment of intervertebral disc degeneration, we utilized the ssGSEA algorithm to estimate the gene degree of immune cells in the sample and then calculated the correlation between key genes and immune cells according to the “Spearman” method. Subsequently, we displayed it through the correlation heatmap (**Figure 10A**), along with the correlation between genes with significant correlation and cells shown in the correlation scatter diagram. There is a significant positive correlation between neutrophils and NOTCH1 (r=0.84, p<0.01, **Figure 10B**), SP1 (r=0.76, p<0.01, **Figure 10C**), and STAT3 (r=0.74, p<0.01, **Figure 10D**), while effector memory CD4 T cells were significantly negatively correlated with NOTCH1 (r=-0.66, p<0.01, **Figure 10E**), RCOR2 (r=-0.62, p<0.01, **Figure 10F**), and SP1 (r=-0.59, p<0.01, **Figure 10G**).

### 3.11 Validation of the hub genes

**Figure 11:**
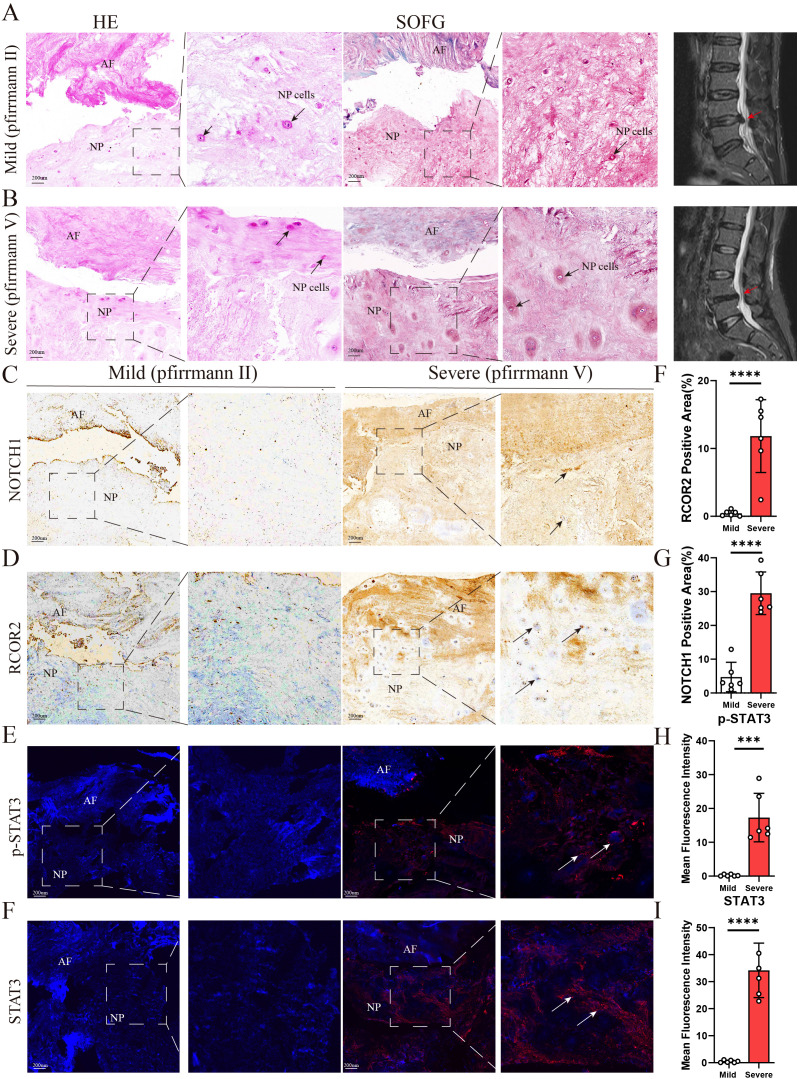
**(A,B)** Representative images of sagittal lumbar spine MRI and histological staining of intervertebral disc samples from the two groups (n = 6). H&E staining and SOFG staining were performed to evaluate structural changes in the NP, AF. The arrows indicate NP cells. **(C-I)** Representative images of IHC and IF staining for NOTCH1, ROCR2, p-STAT3, and STAT3 in intervertebral disc samples from the two groups (n = 6). The arrows indicate positively stained regions. NP: nucleus pulposus; AF: annulus fibrosus. The scale bar indicates 200 μm. Data are presented as means ± SDs. ns, not significant; *p < 0.05; **p < 0.01; ***p < 0.001; ****p < 0.0001.

To validate the identified hub genes, we obtained RNA from 8 intervertebral disc tissues, including 4 in the Mild Group and 4 in the Severe Group. In severe disc degeneration, RCOR2, STAT3, NOTCH1, SP1, and SART1 expression were elevated, while PRIM1 and LYAR expression levels were significantly reduced. However, MSH2 and KIF20B did not show statistically significant differences in PCR analysis (**Supplementary Figure 1E**). To further validate the results from RF and ROC curve analyses, we performed immunofluorescence staining to detect the expression levels of RCOR2, p-STAT3, STAT3, and NOTCH1 in intervertebral disc (IVD) tissues (n = 6). The results showed that RCOR2, STAT3, p-STAT3, and NOTCH1 expression levels were higher in the IDD group compared to controls, with particularly high expression in the severe degeneration group (**Figure 11**).

## 4 Discussion

IDD is a common age-related degenerative condition characterized by nucleus pulposus cell apoptosis, degradation, and metabolic imbalance within the extracellular matrix (ECM) (22–24). The degeneration process, associated with decreased proteoglycan and type I collagen levels, is influenced by genetic factors, mechanical stress, and aging. Current therapeutic strategies are primarily symptomatic, often failing to halt disease progression, while surgical replacement imposes significant economic burdens (25). Recent multi-omics analyses have revealed critical insights into the hypoxia-related molecular mechanisms underlying IDD (26, 27). Additionally, building on previous findings, we identified key hypoxia-related genes, including STAT3, RCOR2, and NOTCH1, which demonstrate diagnostic accuracy through ROC analysis and highlight their potential for early detection of IDD. The discovery of distinct molecular subtypes of IDD suggests varying disease progression pathways, further emphasizing the importance of understanding immune cell infiltration—particularly the roles of macrophages and neutrophils—in disease progression. This knowledge lays a theoretical foundation for immune-centered intervention strategies, opening promising avenues for targeted therapies aimed at modulating ECM degradation pathways.

This study investigates the role of hypoxia-related genes in the pathophysiology of IDD, elucidating their critical involvement in disease progression. Initially, we executed batch effect correction and normalization procedures on the GSE150408 and GSE124272 datasets, successfully establishing a consolidated dataset comprising 25 IDD samples and 25 control samples. Differential expression analysis revealed that 330 genes exhibited statistically significant variations between the IDD cohort and the control group. GO and KEGG enrichment analyses indicated that these genes are predominantly associated with biological processes such as protein ubiquitination and ribosomal biogenesis. Through comparative analysis with hypoxia-related genes, we identified 9 HRDEGs, specifically RCOR2, STAT3, NOTCH1, SP1, SART1, PRIM1, LYAR, KIF20B, and MSH2, which may play pivotal roles in the etiology of IDD. A robust positive correlation was detected between STAT3 and NOTCH1, while significant negative correlations were established between PRIM1 and NOTCH1, as well as between MSH2 and STAT3, indicating a complex interaction network among hypoxia-related genes. Furthermore, the construction of a PPI network elucidated the interactions among STAT3, NOTCH1, and SP1, offering deeper insights into the molecular mechanisms underlying IDD. Notably, CIBERSORT analysis of immune cell infiltration demonstrated a predominance of neutrophils in IDD samples, which were significantly correlated with specific hypoxia-related genes (e.g., NOTCH1, SP1, and STAT3), suggesting a substantial role for neutrophils in the inflammatory immune microenvironment associated with IDD. These findings underscore the influence of the hypoxic microenvironment and inflammatory immune landscape on IDD, providing novel insights into its pathogenic mechanisms. Notably, RCOR2, STAT3, and NOTCH1 exhibited enhanced diagnostic efficacy, highlighting the relevance of HRDEGs in the identification of IDD patients and presenting new therapeutic targets for early diagnosis and intervention.

During IDD progression, ECM degradation significantly affects disc structure, particularly reducing type II collagen and proteoglycan levels, which contributes to calcification and subsequent NP cell death (28, 29). This decline in ECM integrity not only hinders NP cell proliferation but also exacerbates degeneration. Oxidative stress is a key driver in IDD; excessive reactive oxygen species (ROS) induce cellular oxidation and stress, leading to apoptosis and accelerating degeneration (30, 31). ROS modulates NP cell senescence, apoptosis, autophagy, and a pro-inflammatory phenotype, further compromising disc health. Immunological responses also play a significant role. When the blood-NP barrier is compromised, NP cells encounter immune cells, sparking an inflammatory response. The infiltration of these immune cells, which release pro-inflammatory cytokines, intensifies inflammation within the disc, often correlating with increased pain in patients (32–34). In our study, RCOR2 expression was notably elevated in severely degenerated NP cells compared to mild cases, RCOR2, or REST Corepressor 2, is primarily known as a transcriptional co-repressor involved in gene silencing, especially genes linked to neural development and differentiation. RCOR2 is thought to influence IDD by participating in pathways that regulate inflammation, cellular apoptosis, and matrix remodeling in the NP cells (35, 36). Hypoxia, a common condition in the disc microenvironment, may induce RCOR2 expression and impact cellular responses to stress, potentially exacerbating degeneration, suggesting its involvement in oxidative stress and inflammation within IDD. Overall, our research suggests that RCOR2 is a hypoxia related gene with diagnostic potential.

Moreover, our findings indicate that STAT3 and NOTCH1 function as critical regulators in cellular mechanisms that substantially influence the pathogenesis of IDD, particularly within the inflammatory and hypoxic milieu associated with disc herniation. STAT3 is a transcription factor primarily activated by cytokines and growth factors, such as IL-6, and is crucial for regulating inflammation, cell proliferation, and apoptosis (37, 38). In IDD, the hypoxic and inflammatory disc environment may trigger STAT3 activation, which then promotes the expression of inflammatory cytokines, amplifying the local inflammatory response. This creates a feedback loop where inflammation promotes STAT3 activity, which in turn sustains the inflammatory and catabolic processes, further damaging the ECM of the disc. Moreover, STAT3 activation in a hypoxic environment can lead to cellular changes that support NP cell apoptosis and senescence. Under hypoxia, STAT3 may interact with hypoxia-inducible factor 1-alpha (HIF-1α) to regulate genes that respond to oxidative stress and nutrient deprivation, both of which are implicated in disc cell degeneration and apoptosis (39, 40). NOTCH1 is a receptor involved in cell fate determination, differentiation, and apoptosis. In IDD, NOTCH1 signaling is likely to influence NP cell differentiation, chondrocyte hypertrophy, and ECM degradation, contributing to the breakdown of disc integrity. Studies suggest that elevated NOTCH1 expression under hypoxic conditions leads to dysregulated cell differentiation pathways, further exacerbating disc degeneration. Additionally, NOTCH1 signaling is implicated in promoting inflammatory responses within disc tissue. When activated by hypoxia or inflammatory cytokines, NOTCH1 enhances the secretion of pro-inflammatory mediators, which can attract immune cells to the disc, amplifying inflammation and subsequent degeneration (41, 42). Therefore, in IDD, NOTCH1 signaling is likely to influence NP cell differentiation, chondrocyte hypertrophy, and ECM degradation, contributing to the breakdown of disc integrity.

Although RCOR2, STAT3, and NOTCH1 have been implicated in various other diseases, their roles in IDD may involve unique mechanisms driven by the distinct hypoxic and inflammatory microenvironment of degenerating discs. RCOR2 acts as a hypoxia-inducible transcriptional corepressor, suppressing the expression of inflammatory genes such as IL-6 and IL-1β in neural and adipose tissues (43, 44). Conversely, its dysfunction has been linked to enhanced cytokine production and neurodegeneration. STAT3, extensively involved in tumorigenesis and immune dysregulation, can cooperate with HIF-1α under hypoxic stress to modulate angiogenesis and oxidative responses. Similarly, NOTCH1 regulates cellular differentiation and inflammatory cytokine secretion in multiple pathological contexts, including cancer and autoimmune diseases (45, 46). However, the persistent hypoxia, biomechanical loading, extracellular matrix degradation, and immune infiltration that characterize the IDD microenvironment appear to uniquely activate these signaling cascades. Accumulating evidence demonstrates upregulated STAT3 phosphorylation and enhanced NOTCH1 expression in degenerative disc tissues, supporting their specific involvement in IDD. These context-dependent mechanisms underscore the potential specificity of these biomarkers for IDD diagnosis and suggest promising therapeutic targets tailored to the degenerative disc milieu.

One limitation of this study is the lack of compartment-specific analysis of the intervertebral disc. The disc consists of the NP, AF, and CEP, each with distinct cellular populations, matrix components, and responses to mechanical and inflammatory stimuli. Our bulk-level analysis integrates signals from these regions, potentially masking compartment-specific differences in hypoxia-induced pathways. For example, the HIF-1α/Notch1 axis is particularly relevant to NP cell matrix maintenance, whereas inflammatory pathways such as IL-6/STAT3 or RCOR2/NF-κB may be more broadly active across multiple compartments. Future studies employing single-cell RNA sequencing, spatial transcriptomics, or targeted sampling of individual compartments will help to delineate region-specific molecular alterations further and clarify the compartmental contributions to IDD pathogenesis.

Overall, our study provides important insights into the immune microenvironment of IDD, highlighting the role of immune cell infiltration and its association with hypoxia-related hub genes. The significant positive correlations of M0 macrophages with naive CD4^+^ T cells and activated mast cells suggest a cooperative interaction that may sustain inflammation within the degenerative disc. M0 macrophages, known for their potential to polarize into different phenotypes, appear to interact with these immune cells to create a complex inflammatory milieu. Conversely, the observed negative correlations between monocytes and both M0 macrophages and naive CD4^+^ T cells may indicate a competitive or transitional relationship, where monocytes differentiate into macrophage subtypes in response to local inflammatory cues (47, 48). Additionally, the hypoxia-related genes NOTCH1, STAT3, and SP1 showed strong positive correlations with neutrophils, underscoring a potential role in amplifying neutrophil-driven inflammatory responses under hypoxic conditions, which could accelerate tissue degeneration in IDD through the release of ROS and proteolytic enzymes. The negative correlations of these hub genes with effector memory CD4^+^ T cells suggest a dampening of adaptive immune responses, further disrupting immune regulation within the IDD microenvironment. Together, these findings reveal a multifaceted immune response in IDD, with distinct immune cell populations and hypoxia-related genes likely contributing to disease progression. These insights point to potential therapeutic strategies targeting specific immune cells or hypoxia-related genes to mitigate inflammation and slow IDD progression.

## 5 Conclusion

**Figure 12:**
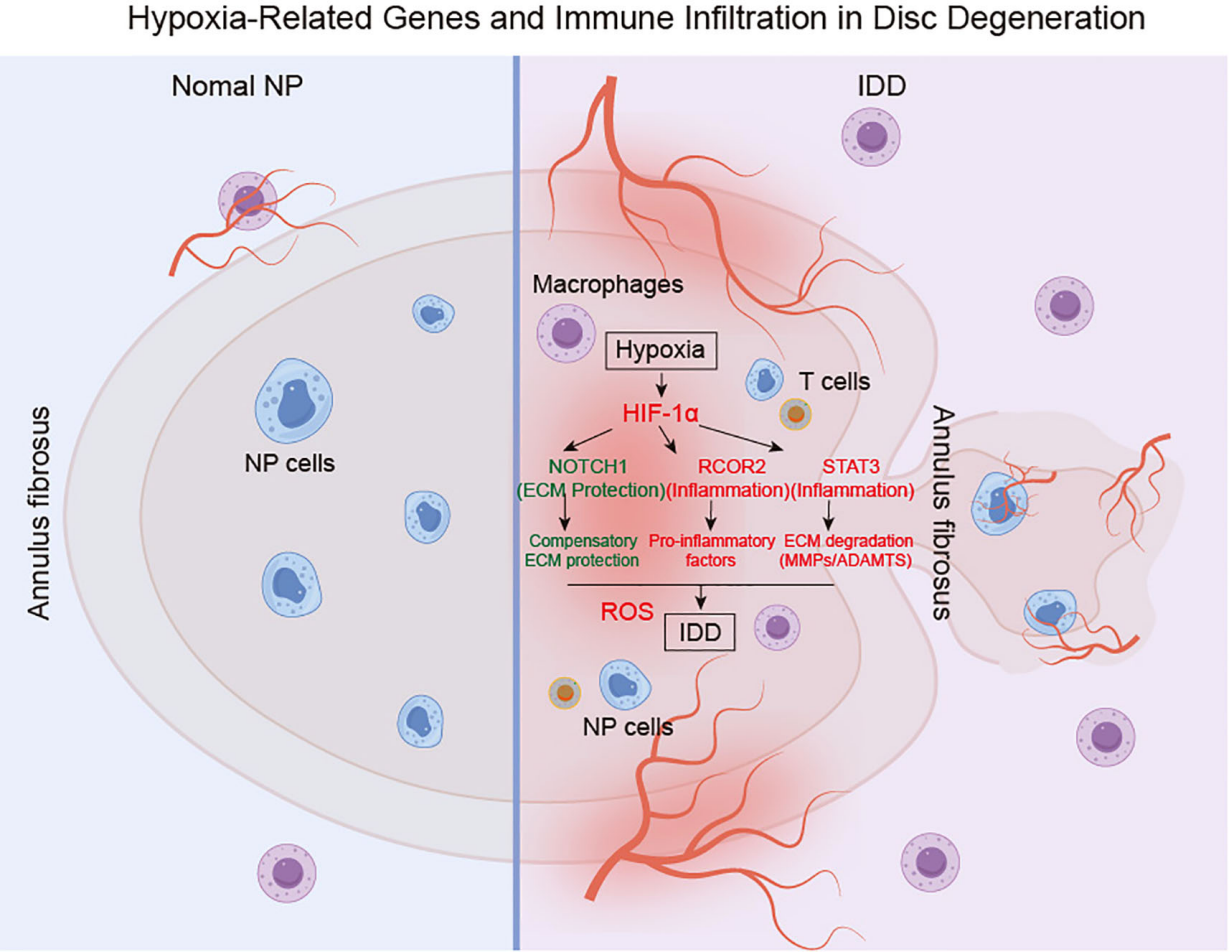
Proposed the regulatory mechanism of hypoxia-associated molecular mechanisms in the IDD. IDD: intervertebral disc degeneration. NP: Nucleus pulposus.

In conclusion, this study elucidates the pivotal roles of hypoxia-related genes, notably RCOR2, STAT3, and NOTCH1, in the pathophysiology of IDD and highlights their diagnostic potential. We demonstrate that STAT3 and NOTCH1 orchestrate pro-inflammatory cytokine regulation, creating a feedback loop that accelerates the deterioration of disc integrity, while RCOR2 exacerbates degeneration by modulating apoptosis and oxidative stress responses (**Figure 12**). Collectively, these findings reveal a complex interplay between hypoxia, immune cell infiltration, and degenerative pathways in IDD, suggesting novel therapeutic avenues for targeted intervention.

## Data Availability

The datasets presented in this study can be found in online repositories. The names of the repository/repositories and accession number(s) can be found in the article/**Supplementary Material**.

## Supplementary material

Supplementary Figure 1 Data preprocessing. **(A, B)** Standardization of merged dataset. Boxplots illustrate the standardization processes of the merged dataset before **(A)** and after **(B)** treatment. **(C, D)** Batch effect treatment. PCA plots depict the Merged dataset before **(C)** and after **(D)** batch effect treatment. IDD: Intervertebral Disc Degeneration; PCA: Principal Component Analysis. **(E)** The gene expression of hub genes in the mild and severe IDD tissues (n = 4). Data are presented as the means ± SDs. ns, no significance; *p < 0.05; **p < 0.01; ***p < 0.001.
