# Supplementary figures and images for: Role of hypoxia-related genes and immune infiltration in intervertebral disc degeneration: molecular mechanisms and diagnostic potential
Source: Front Immunol. 2025 Jul 29;16:1606905. doi: 10.3389/fimmu.2025.1606905 (PMC12341000; doi:10.3389/fimmu.2025.1606905)

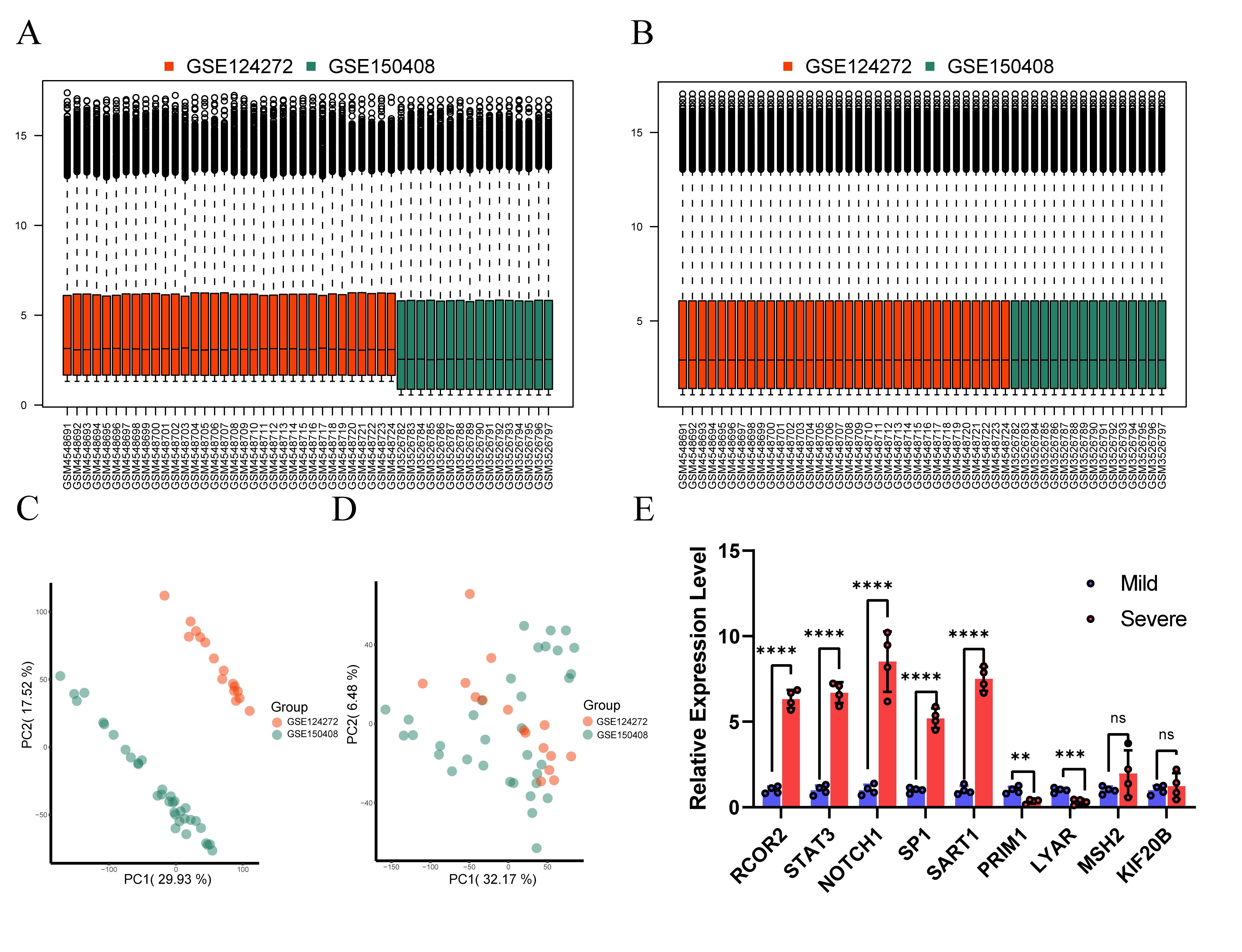

Supplement: Supplementary Figure 1 — Data preprocessing. (A, B) Standardization of merged dataset. Boxplots illustrate the standardization processes of the merged dataset before (A) and after (B) treatment. (C, D) Batch effect treatment. PCA plots depict the Merged dataset before (C) and after (D) batch effect treatment. IDD: Intervertebral Disc Degeneration; PCA: Principal Component Analysis. (E) The gene expression of hub genes in the mild and severe IDD tissues (n = 4). Data are presented as the means ± SDs. ns, no significance; *p < 0.05; **p < 0.01; ***p < 0.001. [file Image1.jpeg]
