# Supplementary material for: Role of hypoxia-related genes and immune infiltration in intervertebral disc degeneration: molecular mechanisms and diagnostic potential
Source: Front Immunol. 2025 Jul 29;16:1606905. doi: 10.3389/fimmu.2025.1606905 (PMC12341000; doi:10.3389/fimmu.2025.1606905)
Supplement: Supplementary file 2 [file Table1.docx]

### **Table S1.** Dataset Information

|  | **GSE150408** | **GSE124272** |
| --- | --- | --- |
| Platform | GPL21185 | GPL21185 |
| Species | Homo sapiens | Homo sapiens |
| Tissue | Whole blood | Whole blood |
| Samples in IDD group | 17 | 8 |
| Samples in Control group | 17 | 8 |
| Reference | Microarray analysis reveals an inflammatory transcriptomic signature in peripheral blood for sciatica. | Transcriptome signatures reveal candidate key genes in the whole blood of patients with lumbar disc prolapse. |

IDD：Intervertebral disc degeneration.
