# Supplementary material for: Role of hypoxia-related genes and immune infiltration in intervertebral disc degeneration: molecular mechanisms and diagnostic potential
Source: Front Immunol. 2025 Jul 29;16:1606905. doi: 10.3389/fimmu.2025.1606905 (PMC12341000; doi:10.3389/fimmu.2025.1606905)
Supplement: Supplementary file 3 [file Table2.docx]

**Table S2: Primers used for RT-qPCR**

| RCOR2 | F:5′- CGAGTGGACAGTAGAGGACAAGGT -3′ |
| --- | --- |
|  | R:5′- TAGTTCGGCTGCGGGTCTTCTT -3′ |
| STAT3 | F:5′- TGGAGAAGGACATCAGCGGTAAGA -3′ |
|  | R:5′- GATAGACCAGTGGAGACACCAGGAT -3′ |
| NOTCH1 | F:5′- AGGACGGCATCAATGGCTTCAC -3′ |
|  | R:5′- GACACAAGGGTTGGATTCACACTCA -3′ |
| SP1 | F:5′- TCAGAACCCACAAGCCCAAACAAT -3′ |
|  | R:5′-GGAGAGTTGAGCAGCATTCACAGT -3′ |
| SART1 | F:5′- AACCTCGCTCTATCCTGTCCAAGT -3′ |
|  | R:5′- CCGCACTACTACCTCCTTCTCCTT -3′ |
| PRIM1 | F:5′- TCGCTACCAATCCTTCAACAACCA -3′ |
|  | R:5′-GTCCTCCTTCAATGCTCTGTCAATG -3′ |
| LYAR | F:5′- GGTGGCAAAGGCTATGAAGGTAAA -3′ |
|  | R:5′- CTTATTGACTGGTTCGCTGTTGGA -3′ |
| MSH2 | F:5′- GGCATATAAGGCTTCTCCTGGCAAT -3′ |
|  | R:5′- CATACCCAACTCCAACCTGTCTCTG -3′ |
| KIF20B | F:5′- ACAGCGAACCATTCAGCAACTC -3′ |
|  | R:5′- GCCAGCCTTTCAACTTCCTCTAAT -3′ |
| GAPDH | F:5′- ACTTTGGTATCGTGGAAGGACTCA -3′ |
|  | R:5′- CCAGTAGAGGCAGGGATGATGTT -3′ |
