# Supplementary material for: Role of hypoxia-related genes and immune infiltration in intervertebral disc degeneration: molecular mechanisms and diagnostic potential
Source: Front Immunol. 2025 Jul 29;16:1606905. doi: 10.3389/fimmu.2025.1606905 (PMC12341000; doi:10.3389/fimmu.2025.1606905)
Supplement: Supplementary file 4 [file Table3.docx]

**Table S3. GO and KEGG enrichment analysis results of differentially expressed genes of different subtypes.**

| ONTOLOGY | ID | Description | p.adjust | qvalue |
| --- | --- | --- | --- | --- |
| BP | GO:0002181 | cytoplasmic translation | 4.5884E-09 | 3.7196E-09 |
| BP | GO:1904667 | negative regulation of ubiquitin protein ligase activity | 0.02417235 | 0.01959525 |
| BP | GO:0031397 | negative regulation of protein ubiquitination | 0.02672949 | 0.02166819 |
| CC | GO:0005840 | ribosome | 5.2435E-12 | 3.7728E-12 |
| CC | GO:0022626 | cytosolic ribosome | 1.1057E-11 | 7.9558E-12 |
| CC | GO:0044391 | ribosomal subunit | 6.776E-10 | 4.8755E-10 |
| MF | GO:0003735 | structural constituent of ribosome | 1.6823E-12 | 1.3132E-12 |
| MF | GO:0055106 | ubiquitin-protein transferase regulator activity | 0.0162845 | 0.01271173 |
| MF | GO:0048027 | mRNA 5'-UTR binding | 0.0180552 | 0.01409395 |
| KEGG | hsa03010 | Ribosome | 8.4349E-13 | 8.0332E-13 |
| KEGG | hsa05171 | Coronavirus disease - COVID-19 | 2.0524E-11 | 1.9546E-11 |
| BP | GO:1903321 | negative regulation of protein modification by small protein conjugation or removal | 0.02974633 | 0.02411378 |
| BP | GO:0051444 | negative regulation of ubiquitin-protein transferase activity | 0.03336677 | 0.02704868 |
| BP | GO:1904666 | regulation of ubiquitin protein ligase activity | 0.03336677 | 0.02704868 |
| CC | GO:0022625 | cytosolic large ribosomal subunit | 1.5319E-07 | 1.1022E-07 |
| CC | GO:0015934 | large ribosomal subunit | 3.4345E-06 | 2.4712E-06 |
| CC | GO:0022627 | cytosolic small ribosomal subunit | 0.00025405 | 0.00018279 |

GO: Gene Ontology; BP: biological process; CC: cellular component; MF: molecular function; KEGG: Kyoto Encyclopedia of Genes and Genomes.
