# Supplementary material for: Role of hypoxia-related genes and immune infiltration in intervertebral disc degeneration: molecular mechanisms and diagnostic potential
Source: Front Immunol. 2025 Jul 29;16:1606905. doi: 10.3389/fimmu.2025.1606905 (PMC12341000; doi:10.3389/fimmu.2025.1606905)
Supplement: Supplementary file 5 [file Table4.docx]

### Table S4. GSEA of different subtypes

| Description | NES | p.adjust | qvalue |
| --- | --- | --- | --- |
| REACTOME_TRANSCRIPTIONAL_REGULATION_BY_TP53 | 1.61257001 | 0.03117291 | 0.02630858 |
| REACTOME_DECTIN_1_MEDIATED_NONCANONICAL_NF_KB_SIGNALING | 1.6993673 | 0.04243411 | 0.03581254 |
| KEGG_OXIDATIVE_PHOSPHORYLATION | 1.57862097 | 0.04243411 | 0.03581254 |
| WP_MAPK_SIGNALING_PATHWAY | -1.54014646 | 0.04981583 | 0.04204239 |
| REACTOME_NEGATIVE_REGULATION_OF_NOTCH4_SIGNALING | 1.86193012 | 0.03117291 | 0.02630858 |
| REACTOME_RRNA_PROCESSING | 2.9149324 | 0.03117291 | 0.02630858 |
| REACTOME_TRANSLATION | 2.88694749 | 0.03117291 | 0.02630858 |
| REACTOME_EUKARYOTIC_TRANSLATION_ELONGATION | 2.88394479 | 0.03117291 | 0.02630858 |
| KEGG_RIBOSOME | 2.86952906 | 0.03117291 | 0.02630858 |
| REACTOME_RESPONSE_OF_EIF2AK4_GCN2_TO_AMINO_ACID_DEFICIENCY | 2.86918465 | 0.03117291 | 0.02630858 |
| REACTOME_EUKARYOTIC_TRANSLATION_INITIATION | 2.84282624 | 0.03117291 | 0.02630858 |
| REACTOME_NONSENSE_MEDIATED_DECAY_NMD | 2.82300717 | 0.03117291 | 0.02630858 |

GSEA: Gene Set Enrichment Analysis
